# Enhanced Thermo–Mechanical Reliability of Ultralow-K Dielectrics with Self-Organized Molecular Pores

**DOI:** 10.3390/ma14092284

**Published:** 2021-04-28

**Authors:** Y. K. Sa, Junghwan Bang, Junhyuk Son, Dong-Yurl Yu, Yun-Chan Kim

**Affiliations:** 1 Advanced Deposition Materials Business Unit, Entegris, Suwon 16229, Korea; 2 Micro-Joining Center, Korea Institute of Industrial Technology, 156 Gaetbeol-ro, Yeonsu-gu, Incheon 406840, Korea; nova75@kitech.re.kr (J.B.); jhson@kitech.re.kr (J.S.); alpha0987@kitech.re.kr (D.-Y.Y.); kyc20@kitech.re.kr (Y.-C.K.); 3 Department of Material Science and Engineering, Korea University, Anam-dong, Seongbuk-gu, Seoul 136713, Korea; 4 Department of Materials Science and Engineering, Seoul National University of Science and Technology, Seoul 01811, Korea

**Keywords:** porous low-k (PLK), SiCOH dielectric thin film, nano-porous structure, Cu/low-k interconnect structure

## Abstract

This paper reported the enhancement in thermo-mechanical properties and chemical stability of porous SiCOH dielectric thin films fabricated with molecularly scaled pores of uniform size and distribution. The resulting porous dielectric thin films were found to exhibit far stronger resistance to thermo-mechanical instability mechanisms common to conventional SiCOH dielectric thin films without forgoing an ultralow dielectric constant (i.e., ultralow-k). Specifically, the elastic modulus measured by nano-indentation was 13 GPa, which was substantially higher than the value of 6 GPa for a porous low-k film deposited by a conventional method, while dielectric constant exhibited an identical value of 2.1. They also showed excellent resistance against viscoplastic deformation, as measured by the ball indentation method, which represented the degree of chemical degradation of the internal bonds. Indentation depth was measured at 5 nm after a 4-h indentation test at 400 °C, which indicated an ~89% decrease compared with conventional SiCOH film. Evolution of film shrinkage and dielectric constant after annealing and plasma exposure were reduced in the low-k film with a self-organized molecular film. Analysis of the film structure via Fourier-transform infrared (FTIR) spectroscopy and X-ray photoelectron spectroscopy (XPS) indicated an increase in symmetric linear Si–O–Si molecular chains with terminal –CH_3_ bonds that were believed to be responsible for both the decrease in dipole moment/dielectric constant and the formation of molecular scaled pores. The observed enhanced mechanical and chemical properties were also attributed to this unique nano-porous structure.

## 1. Introduction

Dielectric thin films with ultralow values of dielectric constant (k), close to or lower than two, have been one of the most intensively investigated materials in recent years for use in on-chip interconnects in high performance ultra-large-scale integrated (ULSI) devices [1,2]. In order to reduce signal interference in ULSI interconnects caused by what is known as the resistance–capacitance (RC) delay, both the resistance of the metal line and the associated capacitance of the interlayer dielectric (ILD) layer should be decreased [3,4]. In general, dielectric constant is defined as the ratio of the capacitance with dielectric material over the capacitance with a vacuum in between the electric plates. Insulating dielectrics with ultralow values of dielectric constant relative to the traditional ILD material (silicon dioxide—SiO_2_) are thus needed to enable current ULSI devices and continued miniaturization to support future integrated products [5,6].

The prevailing approach to lowering the dielectric constant of an ILD material is to add carbon content to SiO_2_, resulting in what is sometimes referred to as a carbon-doped oxide (CDO), but is more accurately described as an organo-silicate (OS) due to the carbon being incorporated primarily as terminal organic (C_x_H_y_) entities [7,8,9,10]. Such materials are nominally SiCOH in composition, with dielectric constant values on the order of 2.8–3.2 [11]. They have been widely adopted for use in ULSI low-k/Cu interconnect structures. However, in order to further reduce the dielectric constant of CDO/OS materials to produce ILDs with ultralow-k values (<2.5), varying levels of nano-porosity have been recently introduced in these materials [12]. The primary method adopted by the microelectronics industry for producing such porous low-k (PLK) materials involves a subtractive approach where a SiCOH matrix precursor is mixed with an organic pore building (i.e., porogen) precursor during plasma enhanced chemical vapor deposition (PECVD). The deposited film is essentially a dual-phase SiCOH-CH_x_ material that is subsequently cured using a combined thermal and ultra-violet (UV) annealing to remove the chemically unstable organic porogen and convert the film into a porous material (p-SiCOH) [13,14,15].

However, p-SiCOH is easily damaged during the wet chemical and plasma-based processing utilized in back-end-of-line (BEOL) interconnect fabrication due to the susceptibility for the terminal organics to be removed from pore surfaces via the reactive chemistries utilized [16,17,18,19]. Such plasma-induced chemical damage becomes especially pronounced as the pore sizes become larger and increasingly interconnected [20]. The damage results in the accumulation of trapped charges and an increase in defect-state concentrations [21,22,23,24]. This leads to large leakage currents and can contribute to a range of other electrical reliability issues [25,26,27]. BEOL process-induced chemical modification of p-SiOCH also contributes to many undesirable physical property deviations that are most prominently manifested via a range of thermal–mechanical reliability failures [28,29,30]. A previous study conducted by Zin et al. clearly showed that exposing p-SiCOH to oxidizing plasmas results in a loss of hydrophobicity, as well as thermo–mechanical stability due to the replacement of terminal CH_3_ groups with silanol (OH) groups [31]. The former leads to increased moisture uptake and significantly increased dielectric permittivity, electrical leakage, and dielectric failure, whereas the latter makes p-SiCOH films prone to delamination, cracking, viscoplastic deformation, and film shrinkage [32,33,34].

Given that plasma-induced damage is difficult to prevent in BEOL processing, one alternative is to chemically repair the process-induced damage [35,36,37]. In accordance with this viewpoint, several methods have been extensively proposed for damage restoration in the past, such as aqueous [38], vapor [39,40] or super-critical CO_2_ enhanced [41,42] chemical silylation processes. A typical silylation process utilizes reactive silyating agents (such as hexamethyldisilazane) to convert hydrophilic Si-OH groups back into hydrophobic Si-CH_3_. However, such restoration processes have shown limited effectiveness or found to work only on the p-SiCOH surface due to the large size of the silylation agents prohibiting penetration into the internal pores.

From these perspectives, a lot of researchers have recently worked on improving the intrinsic chemical and mechanical robustness of the p-SiCOH matrix by structural modification or rearrangement [43,44]. Specifically, Urbanowicz demonstrated that p-SiCOH glasses with improved mechanical properties and ultralow dielectric constant can be obtained by controlled decomposition of the porogen molecules prior to the UV hardening step [45]. Iacopi and Zenasni also showed that the combination of thermal activation and UV radiation promotes a selective bond rearrangement of the organosilicate glass matrix that induces an improvement of about 40% in elastic modulus and hardness value [46,47]. More interestingly, Kikuchi et al. recently reported that a large-radius neutral-beam-enhanced chemical vapor deposition (NBECVD) process can be used to precisely control the film structure so as to produce a non-porous ultralow-k SiCOH with a relatively high modulus (>10 GPa) [48]. Since artificial pores were not formed by this method, the resulting film did not incur any damage from wet (acid or alkali) or oxygen plasma-based BEOL processes [48].

In this study, we build on the work of Kikuchi [48] and others by comparing the thermo–mechanical reliability and the resistance to plasma-induced damage of two different types of p-SiCOH dielectric films, one formed by the subtractive porogen method and the other by a porogenless structural (or constitutive) method. In the structural deposition method, the porosity in the film is obtained by choosing special precursors that contain molecular-sized pores and adjusting the plasma conditions to minimize the dissociation of the precursors in the plasma and promote the polymerization of the precursor molecules. Since the film deposited by the structural method is a single-phase material, the precursor molecules and pores can be uniformly distributed by controlling the deposition condition. Additionally, the post UV or electron beam cure utilized to remove the second phase porogen material in the film becomes unnecessary for the porogenless structural deposition method. Elastic modulus and hardness are specifically investigated for both film types and the resistance to plasma induced damage is also evaluated using the ball indentation method which has been previously shown to be sensitive to integrated BEOL process damage. Our experimental data clearly demonstrate that the thermo–mechanical stability and reliability of an ultralow-k dielectric film can be significantly enhanced through the structural deposition method with special precursors designed to produce a self-organized nano-pore and more uniform pore size distribution without the need for a sacrificial porogen material.

## 2. Experimental Procedure

The porous SiCOH films deposited by the subtractive method in this study were synthesized from an alkoxysilane matrix precursor and an organic porogen (BCHB; bicylcoheptadiene) by PECVD. In case of the film deposited by the structural method, the matrix precursor is confidential and the PECVD condition was tuned from the industry standard. The films had a nominal thickness of 400 nm, porosity of ~30%, and a dielectric constant of approximately 2.1. Porosity in the subtractive film was created by incorporating the organic BCHB porogen into the film and was later removed by a thermal electron beam cure to form the porous SiCOH layer. The mechanical properties with respect to the different deposition methods were measured at room temperature and compared using nano-indention measurements. A conical tip was found to be more suitable for our thin films. Measurements were carried out with a surface approach velocity of 10 nm/s. Ten points on each film were analyzed and the average and standard deviation were calculated. The first 10% of the film thickness was taken as the indentation depth range to determine elastic modulus and hardness. High frequency dielectric constant and film thickness were measured using a spectroscopic ellipsometer. Three different angles from 65 to 75 degrees were used to calculate the change of light polarization induced by the reflection and transmission at the surface and film/substrate interface.

As a way of quantifying the p-SiCOH mechanical instability, especially viscoplasticity, the ball indentation technique was utilized. In our test equipment, a 20 mm diameter alumina ball with a static weight was placed on top of the p-SiCOH films. The weight was chosen to apply ~100–150 MPa of pressure stress to the film in order to simulate the stress that an ILD would experience during p-SiCOH/Cu integration process. The whole test assembly (the ball with static weight and p-SiCOH film on Si substrate) was housed in a chamber where the temperature and environmental conditions were tightly controlled. The test sequence consisted of first removing trapped moisture in the p-SiCOH films, then degassing the chamber and heating stage followed by applying the static load. Removal of water and degassing was achieved with vacuum and N_2_ gas purge cycle at room temperature without making contact between the ball and p-SiCOH films. The test temperature was set as 400 °C, which is the typical maximum allowed temperature in the BEOL integration process. Ball indentation was performed by bringing the ball into contact with the p-SiCOH film after the target temperature was reached. The indentation surfaces were observed using an optical microscope and quantified using a Wyko model NT9100 optical profilometer equipped with Vision software (VEECO, Plainview, NY, USA). Along with the ball indentation test, nano-indentation was applied to clarify observed changes in the mechanical properties. Changes in chemical bonding configuration and composition were investigated using a transmission Fourier transform infrared (FTIR) spectrometer (Bruker, Billerica, MA, USA) and an X-ray photoelectron spectroscopy (XPS) system (Thermo Fisher Scientific, Waltham, MA, USA). Baseline corrected FTIR spectra were obtained for the p-SiCOH films from 750–3500 cm^−1^ at a resolution of 2 cm^−1^ averaged over 128 scans. Deconvolution fitting of the siloxane (Si-O-Si) backbone peak and binding energy were used for backbone structure characterization.

To compare the chemical stability of the subtractive and structural p-SiCOH films, samples of both were exposed to a thermal anneal process and an oxidative plasma and their change in thickness and dielectric constant were measured. For the thermal anneal, the annealing temperature and time were 400 °C and 1 h, respectively. For the plasma oxidation, the plasma source and power used were O_2_/Ar and 50 W, respectively. The plasma exposure time was fixed at 20 min.

## 3. Results and Discussion

**Figure 1:**
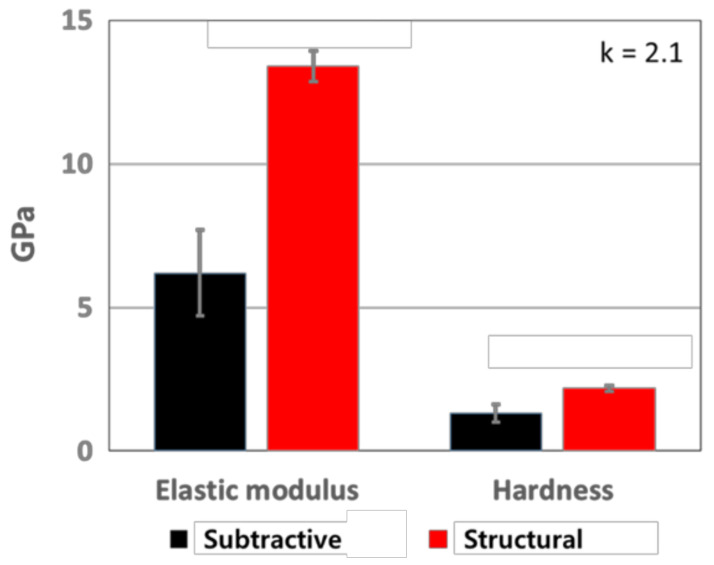
Comparison of elastic modulus and hardness between subtractive and structural p-SiCOH films.

In order to evaluate the change in elastic modulus and hardness of the p-SiCOH films, a series of nano-indentation tests were conducted first on the specimens prepared by the different deposition methods. To minimize the Si substrate effect in the measured values, the indentation depth was controlled to 40 nm. As shown in Figure 1, significant differences in elastic modulus and hardness were observed between the two samples. Specifically, it was found that the p-SiCOH dielectric film deposited by the structural method had enhanced mechanical properties relative to the films deposited by the subtractive method, although dielectric constant remained very low (k ~2.1) for both types of films. In particular, the measured elastic modulus value of 13 GPa for the p-SiOCH film deposited by the structural method is substantially higher than the value of 6 GPa determined for the p-SiOCH film formed by subtractive methods and is also particularly high relative to those previously reported for p-SiCOH thin films deposited by other methods but with similar dielectric constant values [49,50]. Correlatively, hardness follows the same trend as elastic modulus where the measured hardness of the structural film was 2.3 GPa and that of subtractive film was 1.2 GPa.

**Figure 2:**
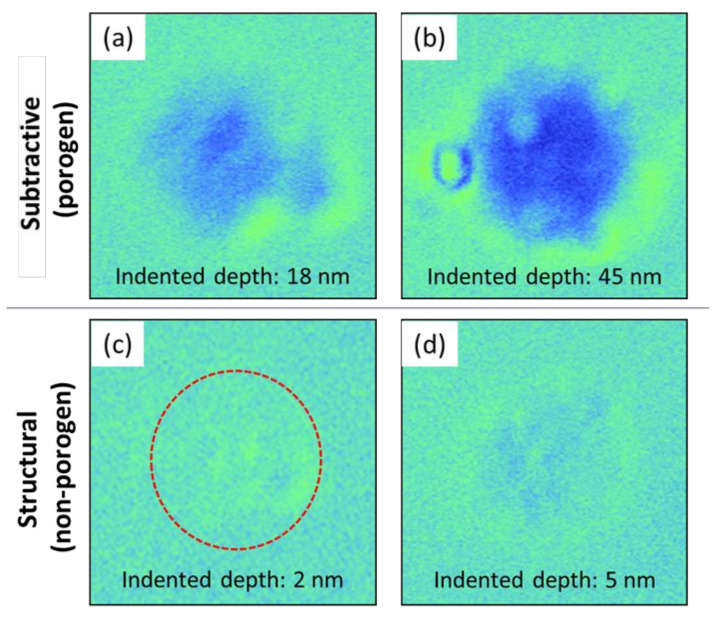
Comparison of viscoplastic deformation between subtractive and structural p-SiCOH films; (**a**,**c**) 1 h and (**b**,**d**) 4 h.

Similar trends were also observed in the ball indentation creep tests. Using this method, we have previously demonstrated evidence of viscosplastic behavior in p-SiCOH thin films formed by primarily subtractive methods. While viscoplasticity has not been considered critical for low-k/Cu interconnects, continued dimensional scaling has resulted in BEOL critical feature sizes now approaching dimensions where viscoplastic deformation may play an important role in interconnect reliability. To illustrate the role that p-SiOCH deposition may play in viscoplastic behavior, Figure 2 presents a series of surface profile images comparing the deformation behavior and indentation depth in the subtractive and structural p-SiCOH films after ball indentation testing. The results clearly show that the degree of deformation by indentation increased with test time (visible as the dark contrast). It is particularly noteworthy that the effect is quite pronounced for the subtractive film while viscoplastic behavior is barely discernible for the structural film when tested using identical conditions. Basically, indented deformation occurs by chemical bond rearrangement via breakage of existing bonds and their re-bonding process. If the film matrix has robust crosslinking among backbone chemical structure and function groups, in other words, the film shows superior mechanical properties, viscoplastic deformation will be diminished. In the previous study on the viscoplastic deformation, some level of viscoplasticity was observed for subtractive films with dielectric constants as high as ~2.7 and porosity levels of ~20% [31]. However, even though the structural film had ~30% porosity and a far lower dielectric constant, it had a much higher resistance to thermo-mechanical stress which could translate to improved thermo-mechanical reliability in an ultralow-k/Cu interconnect. This indicates that mechanical properties would not be sacrificed although dielectric constant of the low-k film is decreased by introducing porosity through the structural deposition method.

**Figure 3:**
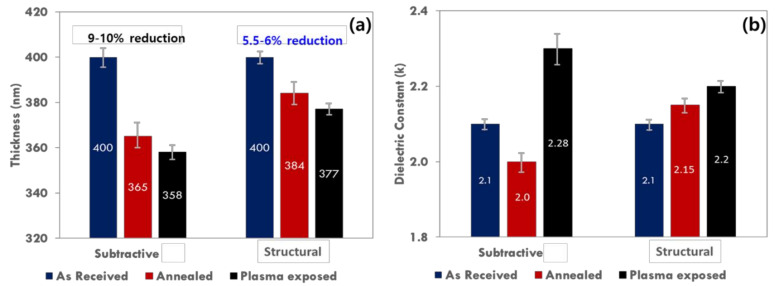
Comparison of the change in dielectric constant and thickness with respect to anneal and plasma exposure between subtractive and structural PLK films. (**a**) Change in deposited film thickness; (**b**) Change in dielectric constant.

To further highlight the enhanced chemical, thermal, and electrical stability of the p-SiCOH films formed by the structural method, Figure 3 provides a comparison for both film types of the change in film thickness and dielectric constant after undergoing a 400 °C, 1 h thermal anneal process or exposure to a 50 W O_2_/Ar plasma for 20 min. Both tests are key metrics for evaluating the potential successful implementation of a new ultralow-k dielectric in BEOL processing. As shown in Figure 3a, the thickness reduction for the subtractive film after thermal annealing was about 10% and that of the structural film was only 4%. Relative to other reports of thermal shrinkage for p-SiOCH films, the 4% thickness reduction is superior. The thickness reduction trend was also observed for films exposed to the O_2_/Ar plasma. This further confirms the greater thermo-chemical stability and resistance to plasma damage for the structural film.

Similar trends in dielectric constant change with plasma exposure were also observed. As shown in Figure 3b, the initial dielectric constant for both films was 2.1 and increased with O_2_/Ar plasma exposure. However, the dielectric constant for the subtractive film increased to 2.3 whereas the dielectric constant for the structural film only increased to 2.2. The dielectric constant increase for both films is attributed to decarbonization of the p-SiCOH dielectrics by the O_2_/Ar plasma exposure. Specifically, terminal Si-CH_3_ bonds are broken by O ions and radicals created in the O_2_ plasma, creating dangling bonds that can then react with ambient moisture to form silanol (Si-OH) groups. The creation of surface silanol groups makes the film surface hydrophilic, and the polarization increase from the moisture uptake and silanol creation, not only results in an increase in dielectric constant, but a reconfiguration of the p-SiCOH network structure as well [51,52].

In contrast, thermal annealing produced a counter trend in the dielectric constant change for the subtractive vs. structural films. As shown in Figure 3b, the dielectric constant for the subtractive film actually decreased to 2.0 after thermal annealing, whereas the dielectric constant for the structural film increased slightly to 2.15. This contrasting behavior is believed to be related to the presence of a porogen residue material (trapped porogen) in the subtractive p-SiCOH dielectric after electron beam curing. Specifically, additional thermal annealing of the subtractive p-SiCOH material results in near complete removal of the remaining labile porogen residue material and subsequently a slight reduction in the dielectric constant. The presence of porogen residues and their influence on the electrical and mechanical properties of subtractively formed p-SiCOH materials has been previously noted by other authors [53,54].

The above contrasting behavior in film shrinkage and dielectric constant change between the subtractive and structurally formed films are largely attributed to differences in the size, size distribution, and interconnectivity of the pores. Generally, porosity incorporation into a dielectric material increases its active surface area, rendering it more susceptible to extrinsic damage from thermal stress and reactive chemicals used in wet chemical and plasma etch and ash processes during BEOL fabrication. Thus, the possibility for chemical and structural damage becomes more significant when the pore size is larger and the pores are more interconnected (i.e., not isolated). Thus, the larger pore size and high pore interconnectivity for the subtractively formed p-SiCOH allows for deeper penetration of high energy oxygen radicals and ions from the plasma into the film matrix. These energetic species consume terminal Si-(CH_3_)_X_ groups on pore surfaces, which induces pore collapse, film shrinkage, and eventually an increase in dielectric constant. However, the structural p-SiCOH film has a smaller pore size and isolated pore distribution. Therefore, thermally-induced stress or plasma modification is restricted primarily to the film surface and thermal/plasma induced damage is reduced. This indicates that the structurally formed dielectric film has superior thermo-mechanical properties and is more likely to withstand the thermally and mechanically-induced stress during the BEOL processing.

**Figure 4:**
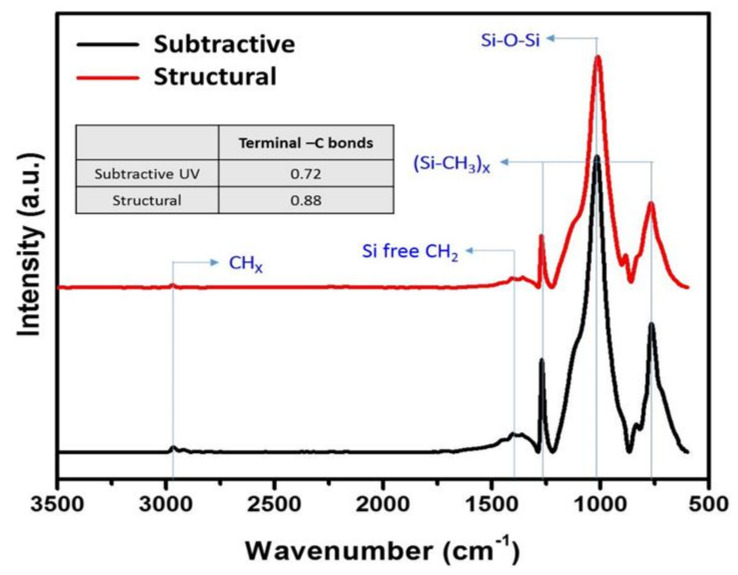
FTIR spectra between subtractive and structural PLK films.

**Figure 5:**
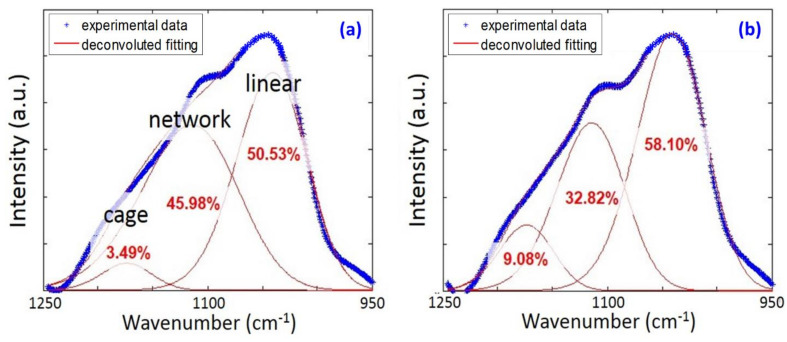
Deconvolution of Si-O-Si backbone peak in FTIR spectra; (**a**) subtractive film and (**b**) structural film.

To fundamentally better understand the chemical bonding and network structure differences between the subtractive and structurally formed p-SiCOH films, transmission FTIR measurements were performed on both the as-deposited films and after the additional thermal anneal and O_2_ plasma exposures. As illustrated in Figure 4 for the as-deposited films, the Si-O-Si asymmetric stretching absorption band at ~1030 cm^−1^ was observed in both cases and represents the network backbone structure for the p-SiCOH dielectrics. The small peak at 1380 cm^−1^ arises from Si-CH_2_-Si linkages, which also represents a small component of the p-SiCOH network backbond structure [55]. Figure 5 presents a detailed deconvolution of the Si-O-Si stretching band into various subcomponents using a Gaussian function with all peaks having the same full-width-half-maximum (FWHM). Specifically, the Si-O-Si bonding configuration was separated into three main subcomponents represented by cage (1135 cm^−1^, Si-O-Si ~150°), network (1063 cm^−1^, Si-O-Si ~140°), and linear or sub-oxide (1023 cm^−1^, Si-O-Si < 140°) structures [55]. According to the area ratio of the fitted sub-peaks, the relative concentrations of all the sub-structures can be estimated. As indicated within Figure 5, this comparison indicates that the cage-like structure is more prominent in the structural film relative to the subtractive film and is indicative of an increased free volume in the film matrix. More specifically, the large Si-O-Si bridging angle for the cage-like structure leads primarily to the formation of nanopores and a reduction in dielectric constant [56]. However, the structural film also exhibited an enhanced concentration of the linear structure (see Figure 5). For NBECVD p-SiCOH films, Kikuchi previously demonstrated that an increased concentration of Si-O-Si linear structures in p-SiCOH can allow both enhanced mechanical properties and reduced dielectric permittivities to be achieved simultaneously. Thus, it can be suggested that the increased linear structure in the structural p-SiCOH film correlates to the enhancement of mechanical properties relative to subtractively formed p-SiCOH film.

The C-H symmetric and asymmetric modes indicative of the terminal CH_3_ organic groups in both p-SiCOH films are also clearly observed at 2700–3000 cm^−1^ in Figure 4. Close inspection of this absorption band additionally shows the presence of C-H_x_ (x = 1–3) stretching modes at 2930 and 2970 cm^−1^ that suggests the possible presence of aliphatic CH_x_ chains [57] In the case of the subtractive film, this may be indicative of trapped or partially decomposed porogen in the film, whereas in case of the structural film, it is indicative of the unreacted precursor. From comparison of these two peaks, the subtractive film appeared to contain a substantial amount of porogen residue, thus supporting the conclusion that the decrease in dielectric constant with thermal annealing for this film was due to the removal of residual porogen.

The FTIR absorption bands centered at 754 and 1260 cm^−1^ in Figure 4, respectively, represent the rocking and deformation vibrational modes for the terminal Si-(CH_3_)_X_ groups in the p-SiCOH films. Higher absorbance for these two modes represents a greater concentration of terminal –CH_3_ groups and in turn more nano-pores in the film matrix. Based on the peak area ratio of these terminal CH_3_ absorption bands to the Si-O-Si stretching band (see table in Figure 4), the structural film appears to have a higher concentration of terminal CH_3_ groups and nanopores relative to the subtractive film. This suggests that the structural film has smaller sized pores since the structural film had more nano-pores while the porosity of two different films was identical [58].

**Figure 6:**
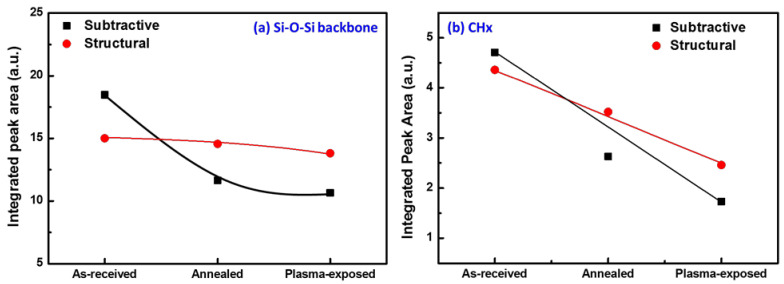
Peak area variations of (**a**) Si-O-Si, and (**b**) CHx after anneal and plasma exposure.

Differences in the chemical structure of the subtractive and structurally formed films were also manifested in the FTIR spectra collected after thermal annealing and O_2_/Ar plasma exposures. Figure 6 specifically shows the change in integrated peak area for the Si-O-Si (950–1250 cm^−1^) and C-H_X_ (2950–3000 cm^−1^) stretching absorption bands in FTIR after thermal annealing and plasma exposure. Focusing first on the change in Si-O-Si absorbance shown in Figure 6a, one can clearly see that the absorbance for the structural film remained relatively unchanged after thermal annealing and O_2_/Ar plasma exposure whereas that for the subtractively formed p-SiCOH film exhibited a sharp drop. This is attributed to Si-O-Si backbone breakage due to either thermal stress during annealing or structural damage induced during O_2_ plasma exposure. Similarly, the C-H_X_ peak absorbance for the subtractive film showed a steeper decline after thermal annealing and O_2_ plasma exposure compared with that of the structural film. As previously discussed, this is consistent with the conclusion that the structural film is less sensitive to BEOL process-induced damage relative to the subtractive film due to the smaller pore size and isolated (low interconnectivity) pore distribution.

**Figure 7:**
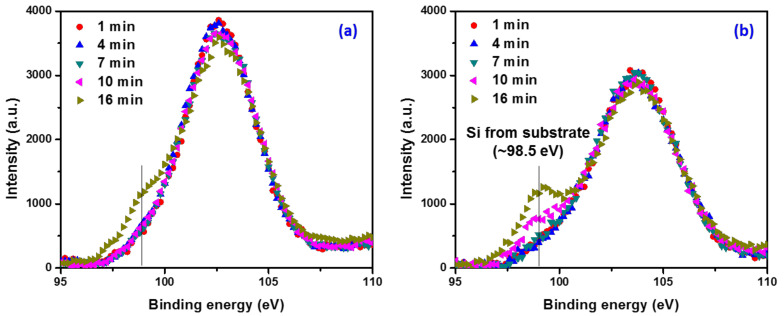
Depth profile of Si atom in vertical direction to the films; (**a**) structural film and (**b**) subtractive film.

To further compare the resistance to BEOL process induced damage, both the subtractive and structurally deposited p-SiCOH films were exposed to an Ar^+^ ion beam sputtering and depth profiling of Si atom was carried out using by X-ray photoelectron spectroscopy (XPS) measurements. Since both SiCOH films were deposited with the same thickness, time-dependent scanning of the XPS Si 2p core level can be utilized to detect when the underlying Si substrate has been reached. If the film matrix has a stronger resistance to ion beam damage, it will take longer for the Ar^+^ ion beam sputter through the p-SiOCH film and reach the Si substrate. For the XPS measurements a monochromatic Al Kα X-ray source was utilized and the Si 2p spectra decomposed into six components: SiO_4_ (104.9 eV), SiO_3_ (103.7 eV), SiO_2_ (102.6 eV), SiO_1_ (101.7 eV), SiO_0_ (100.6 eV) and Si (98.5 eV) where the latter is ascribed to the Si signature from the substrate [59]. As shown in Figure 7, the Si 2p signal at 98.5 eV from the substrate appeared after 10 min sputtering for the subtractive film while the structural film only exhibited the identical signal after 16 min of sputtering. This further supports the greater resistance to plasma damage for the structural film.

**Figure 8:**
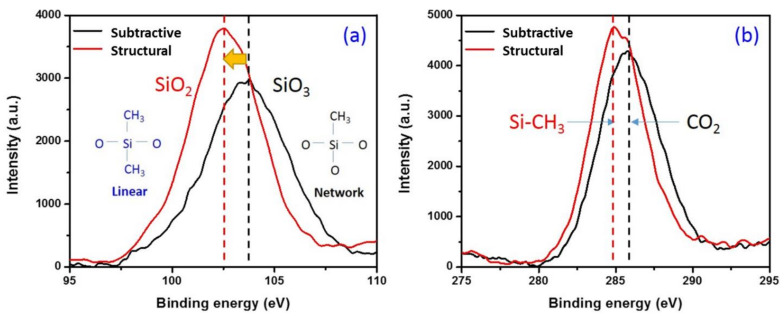
Peak shift of (**a**) Si 2p and (**b**) C 1s.

In summary, thermal–mechanical properties, such as elastic modulus, hardness, and thermally-induced shrinkage, are key indicators for the reliability and performance of p-SiCOH dielectrics in integrated electronic devices. As shown, the structural deposition approach produces films that exhibit enhancements in these properties relative to p-SiCOH films formed by the more traditional subtractive porogen approach. The enhanced properties are largely attributed to the formation of smaller nano-pores with little or no interconnectivity by the structural method relative to the subtractive method. Due to the isolated pore structure, thermally-induced stress and plasma-induced damage are restricted merely to the surface of the dielectric film. This is attributed to the stable siloxane (Si-O-Si) backbone and the terminally bonded methyl group attached to silicon (Si-CH_3_), inducing steric hindrance that lowers the density of the films. As described in Figure 8, Si 2p spectra exhibited the peak shift from SiO_3_ to SiO_2_ (Figure 8a) and C 1s showed more pronounced Si-CH_3_ peak in the structurally deposited film (Figure 8b). The low dielectric constant and superior mechanical stability are closely involved with the formation of the Si-O-Si cage-like structure and an appropriate combination of stable Si-O-Si, Si-CH_3_ groups. Based on the FTIR and XPS spectra, it was concluded that the formation of the Si-O-Si cage-like and linear structures was enhanced by the structural method.

## 4. Conclusions

The robustness of p-SiCOH dielectric materials to withstand thermo–mechanical stress and process-induced damage during BEOL interconnect fabrication is the key to increase the reliability and performance of these materials in microelectronic devices. The present study has clearly shown that the thermo-mechanical properties and resistance to BEOL process induced damage can be significantly improved by use of the structural deposition method. Specifically, the elastic modulus for a structurally formed film was enhanced up to 13 GPa while an ultralow dielectric constant of ~2.1 was achieved. In addition, film thickness shrinkage and dielectric constant increase induced by thermal annealing and O_2_ plasma exposure were remarkably decreased for structurally deposited films relative to standard p-SiCOH films formed using a porogen-based subtractive approach. This implies that p-SiCOH films deposited by the structural method should exhibit reduced integrated damage during BEOL interconnect fabrication.

## Data Availability

Data sharing is not applicable to this article.

## References

[1] Hatton B.D., Landskron K., Hunks W.J., Bennett M.R., Shukaris D., Perovic D.D., Ozin G.A. (2006). Material chemistry for low K-materials. Mater. Today.

[2] Grill A. (2016). PECVD low and ultralow dielectric constant materials: From invention and research to products. J. Vac. Sci. Technol. B.

[3] Treichel H. (2001). Low dielectric constant materials. J. Electron. Mater..

[4] Moors K., Soree B., Tokei Z., Magnus W. (2014). Resistivity scaling and electron relaxation times in metallic nanowires. J. Appl. Phys..

[5] Maex K., Baklanov M., Shamiryan D., Iacopi F., Brongersma S., Yanovitskaya Z. (2003). Low dielectric constant materials for microelectronics. J. Appl. Phys..

[6] King S.W., Simka H., Herr D., Akinaga H., Garner M. (2013). Research Updates: The three M’s (materials, metrology, and modeling) together pave the path to future nanoelectronic technologies. APL Mater..

[7] Volksen W., Miller R., Dubois G. (2010). Low Dielectric Constant Materials. Chem. Rev..

[8] Grill A., Gates S., Ryan T., Nguyen S., Priyadarshini D. (2014). Progress in the development and understanding of advanced low *k* and ultralow *k* dielectrics for very large-scale integrated interconnects State of the art. Appl. Phys. Rev..

[9] Jousseaume V., el Sabahy J., Yeromonahos C., Castellan G., Bouamrani A., Ricoul F. (2017). SiOCH thin films deposited by chemical vapor deposition: From low-κ to chemical and biochemical sensors. Microelectron. Eng..

[10] King S.W. (2014). Dielectric Barrier, Etch Stop, and Metal Capping Materials for State of the Art and beyond Metal Interconnects. ECS J. Solid State Sci. Technol..

[11] Andideh E., Lerner M., Palmrose G., El-Mansy S., Scherban T., Xu G., Blaine J. (2004). Compositional effects on electrical and mechanical properties in carbon-doped oxide dielectric films: Application of Fourier-transform infrared spectroscopy. J. Vac. Sci. Technol. B.

[12] Michalak D.J., Blackwell J.M., Torres J.M., Sengupta A., Kreno L.F., Clarke J.S., Pantuso D. (2015). Porosity scaling strategies for low-*k* films. J. Mater. Res..

[13] Kemeling N., Matsushita K., Tsuji N., Kagami K., Kato M., Kaneko S., Sprey H., de Roest D., Kobayashi N. (2007). A robust k∼2.3 SiCOH low-k film formed by porogen removal with UV-cure. Microelectron. Eng..

[14] Jousseaume V., Zenasni A., Favennec L., Gerbaud G., Bardet M., Simon J.P., Humbert A. (2007). Comparison Between E-beam and Ultraviolet Curing to Perform Porous a-SiOC: H. J. Electrochem. Soc..

[15] Urbanowicz A., Vanstreels K., Verdonck P., van Besien E., Christos T., Shamiryan D., de Gendt S., Baklanov M. (2011). Effect of UV wavelength on the hardening process of porogen-containing and porogen-free ultralow-k plasma-enhanced chemical vapor deposition dielectrics. J. Vac. Sci. Technol. B.

[16] Baklanov M., de Marneffe J.-F., Shamiryan D., Urbanowicz A.M., Rakhimova H.S.T.V., Huang H., Ho P. (2013). Plasma processing of low-k dielectrics. J. Appl. Phys..

[17] Hoofman R.J.O.M., Verheijden G.J.A.M., Michelon J., Iacopi F., Travaly Y., Baklanov M.R., Tokei Z., Beyer G.P. (2005). Challenges in the implementation of low-*k* dielectrics in the back-end of line. Microelectron. Eng..

[18] Hu Q., Kjoller K., Myers A., Singh K.J., King S.W. (2016). Nanoscale chemical structure variations in nano-patterned and nano-porous low-k dielectrics: A comparative photothermal induced resonance and infrared spectroscopy investigation. Vib. Spect..

[19] Rimsza J.M., Du J. (2015). Surface reactions and structural evolution of organosilicate glass under Ar plasma bombardment. Comp. Mater. Sci..

[20] Antonelli G.A., Jiang G., Shaviv R., Mountsier T., Dixit G., Park K.J., Karim I., Wu W., Shobha H., Spooner T. (2012). Synergistic combinations of dielectrics and metallization process technology to achieve 22 nm interconnect performance targets. Microelectron. Eng..

[21] Shamuilia S., Afanas’ev V.V., Somers P., Stesmans A., Li Y.-L., Tokei Z., Groeseneken G., Maex K. (2006). Internal photoemission of electrons at interfaces of metals with low-κ insulators. Appl. Phys. Lett..

[22] Tanbara K., Kamigaki Y. (2010). Paramagnetic Defect Generation and Microstructure Change in Porous Low-k SiOCH Films with Vacuum Baking. J. Electrochem. Soc..

[23] Lauer J.L., Sinha H., Nichols M.T., Antonelli G.A., Nishi Y., Shohet J.L. (2010). Charge Trapping within UV and Vacuum UV Irradiated Low-k Porous Organosilicate Dielectrics. J. Electrochem. Soc..

[24] Bittel B.C., Lenahan P.M., King S.W. (2010). Ultraviolet radiation effects on paramagnetic defects in low-κ dielectrics for ultralarge scale integrated circuit interconnects. Appl. Phys. Lett..

[25] Baklanov M., Zhao L., van Besien E., Pantouvaki M. (2011). Effect of porogen residue on electrical characteristics of ultra low-k materials. Microelectron. Eng..

[26] Pomorski T.A., Bittel B.C., Lenahan P.M., Mays E., Ege C., Bielefeld J., Michalak D., King S.W. (2014). Defect structure and electronic properties of SiOC:H films used for back end of line dielectrics. J. Appl. Phys..

[27] Hussein M.A., He J. (2011). Materials’ impact on interconnect process technology and reliability. IEEE Trans. Semi. Manf..

[28] Volinsky A.A., Vella J.B., Gerberich W.W. (2003). Fracture toughness, adhesion and mechanical properties of low-K dielectric thin films measured by nanoindentation. Thin Solid Films.

[29] Lin Y., Tsui T.Y., Vlassak J.J. (2009). Adhesion Degradation and Water Diffusion in Nanoporous Organosilicate Glass Thin Film Stacks. J. Electrochem. Soc..

[30] Tambat A., Lin H.-Y., Subbarayan G., Jung D.Y., Sammakia B. (2012). Simulations of Damage, Crack Initiation, and Propagation in Interlayer Dielectric Structures: Understanding Assembly-Induced Fracture in Dies. IEEE Trans. Dev. Mater. Rel..

[31] Zin E.H., Bang W.H., Ryan E.T., King S., Kim C.-U. (2013). Study of viscoplastic deformation in porous organosilicate thin films for ultra low-k applications. Appl. Phys. Lett..

[32] Li Y., Ciofi I., Carbonell L., Heylen N., van Aelst J., Baklanov M.R., Groeseneken G., Maex K., Tokei Z. (2008). Influence of absorbed water components on SiOCH low-k reliability. J. Appl. Phys..

[33] Darnon M., Chevolleau T., Licitra C., Rochat N., Zocco J. (2013). Analysis of water adsorption in plasma-damaged porous low-k dielectric by controlled-atmosphere infrared spectroscopy. J. Vac. Sci. Technol. B.

[34] Chang T.C., Chen C.W., Liu P.T., Mor Y.S., Tsai H.M., Tsai T.M., Yan S.T., Tu C.H., Tseng T.Y., Sze S.M. (2003). Moisture-Induced Material Instability of Porous Organosilicate Glass. Electrochem. Sol. Stat. Lett..

[35] Imada T., Nakata Y., Ozaki S., Kobayashi Y., Nakamura T. (2015). Systematic investigation of silylation materials for recovery use of low-*k* material plasma damage. Jpn. J. Appl. Phys..

[36] Bohm O., Leitsmann R., Planitz P., Radehaus C., Schreiber M., Schaller M. (2011). *k*-Restoring Processes at Carbon Depleted Ultralow-k Surfaces. J. Phys. Chem. A.

[37] Forster A., Wagner C., Gemming S., Schuster J. (2015). Theoretical investigation of an in situ k-restore process for damaged ultra-low-k materials based on plasma enhanced fragmentation. J. Vac. Sci. Technol. B.

[38] Fischer T., Ahner N., Zimmermann S., Schaller M., Schul S.E. (2012). Influence of thermal cycles on the silylation process for recovering *k*-value and chemical structure of plasma damaged ultra-low-*k* materials. Microelectron. Eng. April..

[39] Chaabouni H., Chapelon L.L., Aimadeddine M., Vitiello J., Farcy A., Delsol R., Brun P., Fossati D., Arnal V., Chevolleau T. (2007). Sidewall restoration of porous ultra low-k dielectrics for sub-45 nm technology nodes. Microelectron. Eng..

[40] Oszinda T., Schaller M., Schulz S.E. (2010). Chemical Repair of Plasma Damaged Porous Ultra Low-κ SiOCH Film Using a Vapor Phase Process. J. Electrochem. Soc..

[41] Jung J.M., Kwon H.S., Li W.-K., Choi B.-C., Kim H.G., Lim K.T. (2010). Repair of plasma-damaged p-SiOCH dielectric films in supercritical CO_2_. Microelectron. Eng..

[42] Vyhmeister E., Reyes-Bozo L., Valdes-Gonzalez H., Salazar J.-L., Muscat A., Estevez L.A., Suleiman D. (2014). In situ FTIR experimental results in the silylation of low-k films with hexamethyldisilazane dissolved in supercritical carbon dioxide. J. Supercrit. Fluid..

[43] Li H., Knaup J.M., Kaxiras E., Vlassak J.J. (2011). Stiffening of organosilicate glasses by organic cross-linking. Acta Mater..

[44] Krishtab M., de Marneffe J.-F., de Gendt S., Baklanov M.R. (2017). Plasma induced damage mitigation in spin-on self-assembly based ultra low-k dielectrics using template residues. Appl. Phys. Lett..

[45] Urbanowicz A.M., Vanstreels K., Verdonck P., Shamiryan D., de Gendt S., Baklanov M.R. (2010). Improving mechanical robustness of ultralow-k SiOCH plasma enhanced chemical vapor deposition glasses by controlled porogen decomposition prior to UV-hardening. J. Appl. Phys..

[46] Iacopi F., Travaly Y., Eyckens B., Waldfried C., Abell T., Guyer E.P., Gage D.M., Dauskardt R.H., Sajavaara T., Houthoofd K. (2006). Short-ranged structural rearrangement and enhancement of mechanical properties of organosilicate glasses induced by ultraviolet radiation. J. Appl. Phys..

[47] Zenasni A., Jousseaume V., Holliger P., Favennec L., Gourhant O., Maury P., Berbaud G. (2007). The role of ultraviolet radiation during ultralow k films curing: Strengthening mechanisms and sacrificial porogen removal. J. Appl. Phys..

[48] Kikuchi Y., Wada A., Kurotori T., Sakamoto M., Nozawa T., Samukawa S. (2013). Non-porous ultra-low-k SiOCH (k = 2.3) for damage-free integration and Cu diffusion barrier. J. Phys. D Appl. Phys..

[49] Burkey D.D., Gleason K.K. (2004). Organosilicon Thin Films Deposited from Cyclic and Acyclic Precursors Using Water as an Oxidant. J. Electrochem. Soc..

[50] Rathore J.S., Interrante L.V., Dubois G. (2008). Ultra Low-k Films Derived from Hyperbranched Polycarbosilanes (HBPCS). Adv. Funct. Mater..

[51] Kubasch C., Klaus C., Ruelke H., Mayer U., Bartha J.W. (2010). Investigation of Moisture Uptake in Low-κ Dielectric Materials. IEEE Trans. Electron Dev..

[52] Kubasch C., Olawumi T., Ruelke H., Mayer U., Bartha J.W. (2014). Erratum: Investigation of Argon Plasma Damage on Ultra Low-κ Dielectrics. ECS J. Solid State Sci. Technol..

[53] French B.L., King S.W. (2013). Detection of surface electronic defect states in low and high-k dielectrics using reflection electron energy loss spectroscopy. J. Mater. Res..

[54] Stan G., Gates R.S., Kavuri P., Torres J., Michalak D., Ege C., Bielefeld J., King S.W. (2014). Mechanical property changes in porous low-k dielectric thin films during processing. Appl. Phys. Lett..

[55] Grill A., Neumayer D.A. (2003). Structure of low dielectric constant to extreme low dielectric constant SiCOH films: Fourier transform infrared spectroscopy characterization. J. Appl. Phys..

[56] Grill A., Patel V. (2001). Ultralow-k dielectrics prepared by plasma-enhanced chemical vapor deposition. Appl. Phys. Lett..

[57] King S.W., French M., Bielefeld J., Lanford W.A. (2011). Fourier transform infrared spectroscopy investigation of chemical bonding in low-k a-SiC:H thin films. J. Non-Cryst. Sol..

[58] Oh K.S., Choi C.K. (2004). Nano Pore Structure of Low-k SiOC(-H) Films Measured by Small Angle Neutron Scattering. J. Korean Phys. Soc..

[59] Wang W., Grozea D., Kim A., Perovic D.D., Ozin G.A. (2009). Vacuum-Assisted Aerosol Deposition of a Low-Dielectric-Constant Periodic Mesoporous Organosilica Film. Adv. Mater..

